# Comparative Analysis of Screw Loosening between SynOcta and InOcta Tissue Level Abutments: An *In Vitro* Study

**DOI:** 10.1055/s-0045-1808257

**Published:** 2025-05-07

**Authors:** Amirhossein Fathi, Erfan Esmaeilian, Sepideh Salehi, Ramin Mosharraf, Ramin Atash

**Affiliations:** 1 Department of Prosthodontics, Faculty of Dentistry, Isfahan University of Medical Sciences, Isfahan, Iran; 2 Department of Dentistry, Faculty of Dentistry, Isfahan University of Medical Sciences, Isfahan, Iran; 3 Department of Dental Prosthodontics, Université Libre de Bruxelles, Brussels, Belgium

**Keywords:** abutment, screw loosening, InOcta abutment, SynOcta abutment

## Abstract

**Objective:**

Implants are one of the common treatments in dentistry. This treatment has various complications such as inflammation around the implant, failure of the coating, and screw loosening. Several factors contribute to screw loosening, including abutment type and collar height. Therefore, this study aims to compare the amount of loosening in two types of abutments—InOcta and SynOcta abutments.

**Materials and Methods:**

In this laboratory study, 20 titanium fixtures of the Dentis brand were divided into two groups. Each group consisted of 10 fixtures. The fixtures were mounted vertically in acrylic blocks with dimensions of 20 × 6 × 10 mm. After installing the SynOcta and InOcta abutments, the screws were torqued to 30 N·cm and re-torqued after 10 minutes. Subsequently, the samples were transferred to a chewing simulator. A compressive force of 90 N was applied for 10,000 cycles at a frequency of 75 rpm. After loading, the torque required to loosen the screws was measured, and the loosening torque was calculated. The data were analyzed using an independent *t* -test, and a significance level ( *p* -value) of less than 0.05 was considered.

**Results:**

The mean de-torque for the tissue level InOcta abutments was calculated to be 25.75 N.cm, while the mean de-torque for the SynOcta abutments was 21.98 N.cm. A comparison using the *t* -test showed that the mean de-torque for the InOcta abutment group was significantly higher than the SynOcta group ( *p* < 0.001).

**Conclusion:**

The final results of the experiments indicate that under laboratory conditions, the de-torque of the abutment screw in the tissue level SynOcta group is significantly lower than that in the InOcta group ( *p* < 0.001).

## Introduction

Abutment screw loosening is a critical concern in dental prosthetics. It significantly affects the stability and useful life of prostheses. 1 The entry of body fluids into the dental implant assembly lowers the preload value, causing the tightening torque of the dental implant abutment to be lost. 2 Internal hexagonal abutment connections and CFR-PEEK composite materials are exceptionally good at preventing screw loosening. 3 Addressing this issue through proper torque management, anti-rotation features, and retightening protocols is crucial for the longevity and success of implants. 4 Torque loss in abutment screws varies, with the MTC model consistently experiencing high torque loss, while the IHC model shows minimal loss. 5 Decreasing the abutment screw pitch can also be an effective method to increase resistance to screw loosening. 6 InOcta and SynOcta abutments, two common types of abutments in dental prostheses, have different characteristics. The InOcta system is designed with a smooth surface for immediate or early loading, promoting quicker healing. The SynOcta system features a unique octagonal connection, which enhances stability and facilitates a precise fit between the implant and the abutment. This study compares screw loosening in InOcta and SynOcta abutments through *in vitro* testing. This study can help to improve the performance and quality of dental prostheses and provide useful guidelines for selecting the appropriate abutment in dental prostheses. 7 8 A dental implant is an alloplastic object, material, or other tissue partially or completely placed or transplanted into the body for therapeutic, diagnostic, prosthetic, or clinical testing purposes. 9

Dental implants are known as an appropriate alternative treatment for tooth loss, although this treatment can also be associated with problems. 10 Many studies have recently reported the occurrence of many problems and complications after implant treatment. For example, potential complications such as failure of osseointegration, surgical complications, marginal bone loss, inflammation around the implant site, mechanical complications, and cosmetic problems have been observed. 11 Additionally, mechanical complications have been reported, including screw loosening, screw fractures, implant fractures, veneer fractures, and reduced attachment in implant-supported dentures. 12

Screw loosening is one of the common complications in implant treatment. 13 14 15 A study showed that screw loosening was the most common problem that occurred during the first year of treatment in 107 cases of one-unit implant restorations using the Per-Ingvar Brånemark system. 13 Another study examined patients with implant treatment for 5 years and showed that screw loosening occurred in 7.6% of cases. 14 The screw connects the abutment to the implant. When a screw is tightened, a rotational force is applied to it, while a tensile force is produced by stretching. This creates a “clamping force” that maintains the implant–abutment connection (preload). 16 However, the screw can loosen in the presence of a load higher than the clamping force or when preload is lost. 17 When this happens, the abutment and implant can become mobile, affecting the surrounding soft tissue and the implant structure, and potentially leading to local inflammation. Additionally, when stress is concentrated, it may result in screw fracture, abutment fracture, or even implant fracture. 18 19 20 Various factors including the implant type and design can affect this component. 21 22 The Dentis implant system is one of the most common implant systems used in Iran. 23 In this system, two types of abutments SynOcta and InOcta are made (these abutments were chosen because they are widely used), which are different from each other in internal geometry. Thus, this study investigates the effect of these two types of abutments on the rate of screw loosening.

## Materials and Methods

This study was conducted in 2024 in the Faculty of Dentistry of the University of Medical Sciences. The data were collected in the form of laboratory samples. In this laboratory study, donated filled fixtures were used, which were fully evaluated in terms of chemical corrosion, mechanical wear, and fracture before starting the experiment to ensure the validity of the research results. Specifically, 20 Dentis brand titanium-filled fixtures were selected and divided into two groups including 10 fixtures for SynOcta abutments and 10 fixtures for InOcta abutments. So, the sample size was 10. 24 In each fixture, SynOcta and InOcta abutments were mounted respectively.

For each implant, conical tissue level fixtures and titanium screws were used. The investigated implant systems were mounted on acrylic blocks with dimensions of 20 × 6 × 10 mm perpendicular to the surface (20 = height,10 = length, 6 = width). The vertical position of the implant inside the resin blocks was fixed by the supervisor, and the abutments were mounted on the fixture. After mounting the abutments in their respective fixtures, the screws were tightened to a torque of 30 N/cm using a Cedar DID-4 digital torque driver (made in Japan), following the manufacturer's instructions. After 10 minutes, the screws were re-tightened to the same torque due to torque loss occurring after the initial tightening. 25 26 Then, the samples and resin blocks were transferred to the Chewing Simulator Machine. In this step, a compressive force equivalent to 90 N was applied to the samples and inserted into the center of each sample for 10,000 cycles with a frequency of 75 rpm (which is equivalent to the number of times a person chews in a year). After the end of the loading period, the screws were loosened once more, and the torque required to loosen them was measured. The rate of loosening was calculated. After measuring and recording the screw loosening forces using *t* -test analysis, the results were compared at the error level of 0.05 using SPSS26 software. Since the study was not conducted on humans, there were no special ethical considerations required for the implementation of the project.

## Results

A comparison was made using the *t* -test after ensuring the normality of the data in two groups using the Kolmogorov–Smirnov test (with a significance level of 5%). The results revealed that the mean de-torque score of the abutment screw in the SynOcta tissue level group was significantly lower than that in the InOcta tissue level group ( *p* < 0.001). Based on Table 1 , among 10 tissue abutment samples of InOcta, the rate of de-torque was measured in the range of 25.1 to 26.4 N/cm. The mean de-torque in this group was 25.75 N/cm and the median was 25.8 N/cm. The standard deviation of de-torque was 0.46 N/cm and the 95% confidence interval was calculated between 26.07 and 25.42 N/cm ( Table 1 ).

**Table 1:** Investigating the amount of de-torque in N/cm in two groups of InOcta and SynOcta tissue level abutment groups

Abutment type	Min		Max	Mean	Median	SD	Confidence interval	*t*	df	Sig.
InOcta tissue level abutment	25.0	26.4	25.75	25.8	0.46	25.42 26.07	25.1	11.52	18	>0.001
SynOcta tissue level abutment	20.4	23.7	21.98	22.05	0.93	21.31 22.64	20.4			

For SynOcta group abutments, the amount of de-torque was recorded from 20.4 to 23.7 N/cm. The mean de-torque was 21.98 N/cm and the median was 22.05 N/cm. The standard deviation of de-torque was 0.93 N/cm and the 95% confidence interval for its mean was between 21.31 and 22.64 N/cm. The results of the *t* -test comparing the mean de-torque between the two groups indicated that the T-statistic value was 11.52, with 18 degrees of freedom (df).

**Fig. 1:**
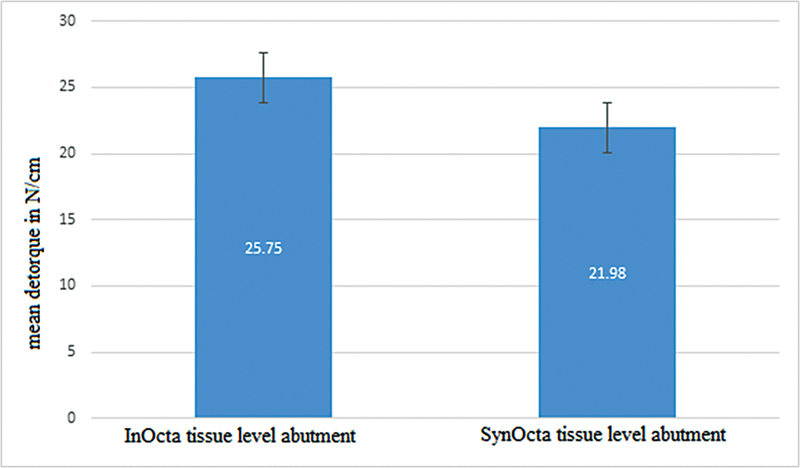
Comparison of the mean de-torque in the two groups of InOcta and SynOcta tissue level abutment in N/cm.

The significance level ( *p* -value) was less than 0.001, indicating a statistically significant difference between the two groups ( Fig. 1 ).

## Discussion

The abutment screw is a crucial component in the connection between the implant and the abutment in most implant systems. During tightening, the screw experiences elastic deformation and stretching, which generates a tensile force known as preload. 17 24 In other words, the screw acts as a tension spring and creates a force that firmly connects the implant and the abutment and keeps these two parts together. 27 Bickford and Oliver have divided the screw-loosening process into two distinct stages. 28 First, the initial tensile deformation of the screw under the influence of the external force is reduced and leads to the reduction of the clamping force. Then, in the second stage, the micro-movement at the junction of the implant and the abutment increases with a further reduction of the clamping force, and this instability leads to screw loosening. 29 Preload reduction caused by external forces is the primary reason for abutment screw loosening. The stability of the connection between the implant and the abutment largely depends on the type of connection used. This issue has been supported by numerous scientific studies. 30

A study by Ahn et al confirms similar results. This study indicates that in external connections, uneven distribution of stress can lead to problems such as breaking screws and reducing the stability of the connection. 31 Internal connection refers to the design through which the abutment is attached to the implant, typically with a structure of 4 to 6 mm. This design effectively increases the contact surface area between the abutment and the implant, improving the distribution of stresses during loading. Consequently, the change in stress distribution enhances the stability and efficiency of the connection. 32 Kofron et al investigated the internal connections of implants and observed that well-constructed internal designs can provide a greater contact surface between the abutment and the implant, leading to more optimal stress distribution and reduced screw tension. This helps to reduce problems such as screw fracture and improve the stability of the connection. 33

Sakamoto et al conducted a study on the implant–abutment connection and found that internal connections have significant advantages over external connections in maintaining stability. The study also indicates that internal connections provide greater resistance to torque reduction and screw loosening. 34 Another study by Segundo et al demonstrated that internal connections significantly improve load distribution and reduce stress compared with external designs. The study specifically emphasized that a more effective design of internal connections offers greater resistance to screw loosening and decreased torque. 35 Given the important role of the abutment in connecting the implant to the restoration, differences in the design of abutments can significantly affect connection stability. Various geometric designs of abutments are likely to alter the stress distribution at the implant–abutment connection point and may also influence the level of torque reduction. 36

Yenigun et al examined the impact of different abutment designs on stress distribution and mechanical stability of the implant–abutment connection. The results revealed that geometric differences in abutment design can significantly change the stress distribution, and affect the stability of the connection, and the level of torque reduction. Different abutment designs have different impacts on the overall stability of the connection by changing the load and stress distribution. 37 In addition to the effect of the geometry of the upper part of the abutment connected to the restoration, the geometric design of the lower part of the abutment that is in contact with the internal implant has a significant impact on reducing the torque and stability of the connection. The rotational freedom between the implant and the abutment is considered a key factor in maintaining the stability of the implant–abutment connection. In the condition that the rotational freedom is less than 2 degrees, the implant–abutment connection will be more stable. However, when the rotational freedom reaches more than 5 degrees, the level of torque reduction increases significantly and this can lead to instability and screw loosening.

These results emphasize the importance of accurate abutment design and limited control of rotational freedom. They also indicate that optimal abutment designs can help to maintain the stability and optimal performance of the connection. The study by Bédouin et al indicated the effect of different abutment designs and rotational freedom on the mechanical stability of the implant–abutment connection. The results indicate that the geometric design of the abutment and the degree of rotational freedom significantly affect the connection stability. In particular, the torque reduction increases dramatically and may lead to instability and screw loosening when the rotational freedom exceeds the desired limit. This study emphasizes the importance of careful abutment design and limiting rotational freedom to maintain optimal performance and connection stability. 38 *In vitro* studies indicate that abutments with conical design show higher resistance to torque loss than other types of abutments, both before and after cyclic loading. This conical design specifically creates a better fit between the abutment and the implant. The result of this improved fit is the reduction of micro-gaps and micro-movements in the implant–abutment connection point, leading to a significant reduction in torque loss. In other words, the conical design has better stability and durability by reducing gaps and unwanted movements in the connection. 39

Sammour et al examined the effect of different abutment designs, including the conical design, on the resistance to torque loss. The results indicated that, unlike other studies, the conical design has a similar performance in torque reduction compared with other designs. This study also provided different results compared with previous studies and indicated that the conical design may not be as effective in reducing micro-gaps and increasing the stability of the connection as some other designs. 40 The results of this study revealed that the InOcta tissue-level abutments show higher stability against screw loosening due to the design and materials used. The independent *t* -test indicated that the mean de-torque score of the SynOcta tissue-level abutment screw was significantly lower than the InOcta tissue-level abutment screw. These results indicate that InOcta tissue-level abutments cause less screw loosening, which can be due to the combination of materials used and their more optimal design.

Additionally, InOcta tissue-level abutments require more attention and care during the treatment to prevent screw loosening, despite their aesthetic benefits. This difference may be due to the differences in the structure and design of these two types of abutments, which can affect the level of stress on the screws. Generally, the results indicate that InOcta tissue-level abutments have a better performance in reducing screw loosening than SynOcta tissue-level abutments due to the design and materials used. This confirms the effect of abutment design on the stability and performance of implants and shows that the correct selection of abutment can play a vital role in the success of dental implant treatments. 41 The study by Pardal-Peláez et al is among the studies conducted in this field. This study examined the impact of abutment design on screw stability and loosening. The results revealed that abutments with proper design and the use of materials with superior mechanical properties significantly reduce screw loosening and provide higher stability under different loading conditions. These results are consistent with those of the present study and emphasize that the design of abutments plays a key role in reducing screw loosening problems and positively affects the overall performance of dental implant treatments. 42

## Conclusion

Based on the experiments, it is concluded that the de-torque of the abutment screw in the SynOcta tissue level group was significantly lower than that in the InOcta group under laboratory conditions ( *p* < 0.001).

## Limitation

The limitations were the small sample size and the period of the cycles during tests was short (∼1 year). More studies should be conducted in different cycles and different implant systems to confirm the results.
